# Chronic anemia due to transmural e-PTFE anti-adhesive barrier mesh migration in the small bowel after open incisional hernia repair: A case report

**DOI:** 10.1016/j.ijscr.2018.10.012

**Published:** 2018-10-12

**Authors:** Francesca Ceci, Linda D’Amore, Elena Annesi, Lucia Bambi, Maria Romana Grimaldi, Francesco Gossetti, Paolo Negro

**Affiliations:** Department of General Surgery "P. Stefanini", Sapienza, University of Rome, Italy

**Keywords:** Mesh migration, Mesh erosion, Anti-adhesive barrier, Mesh-related complications, Case report

## Abstract

**Introduction:**

Meshes are commonly employed in abdominal hernia repair to reduce recurrence rates. Prosthetic repair, however, increases the risk of mesh related complications, including migration into adjacent viscera and erosion which can occur as uncommon and can be difficult to be diagnose.

**Presentation of case:**

This is a case of transmural migration of composite mesh into the bowel, presenting as chronic abdominal pain and anemia 14 years after incisional hernia repair.

**Discussion:**

Mesh implantation in hernia repair has increased the incidence of complications, such as seroma, hematoma and infection. Migration into adjacent viscera and erosion may present as complications related to the use of meshes. Their precise frequency after abdominal wall hernia repair is not well known and their late occurrence can make the diagnosis difficult.

**Conclusion:**

Transmural migration of composite mesh is an uncommon complication of incisional hernia repair. Its pathogenesis is still not completely clear but it has been reported many years after implant surgery. It should be considered in a typical presentation of patients with history of previous prosthetic ventral hernia repair.

## Highlights

• Mesh related unusual complication.
• Intraluminal mesh migration.
• Mesh erosion.

## 1 Introduction

The case report is compliant with the SCARE Guidelines [1]. Incisional hernia is the most common complication of abdominal surgery. Its incidence is 10–15%, with a recurrence rate ranging from 20 to 45%, after anatomic repair [2,3]. Due to this high rate of recurrence, the use of meshes is recommended to reinforce the repair and to reduce tension on the abdominal wall, thus ensuring a "tension-free" reconstruction, which is more resistant to intra-abdominal pressure. Prosthetic surgery, in fact, has significantly decreased recurrence rate from 50% to 10–20% [4]. However, a rise of specific complications has been reported in the literature. Mesh-related complications may occur over years. Among these, mesh migration is an uncommon event resulting from mesh erosion of surrounding structures. Since late presentation of mesh migration is rare, its diagnosis may be challenging.

We report an unusual case of transmural mesh migration of a composite mesh into the bowel, presenting at the OUR INSTITUTION as chronic abdominal pain and anemia, 14 years after incisional hernia repair.

## 2 Case report

A retired 76 year-old Caucasian man, BMI 27, in July 2017 was referred to OUR INSTITUTION with a 2-year history of persistent abdominal pain, resistant to analgesics (Paracetamol and Ketoprofen), irregular bowel habits and rectorrhagia. He reported a 1-year history of iron-deficiency anemia (≈7 g/dl), treated with blood transfusions and investigated with upper and lower endoscopy. The patient had a medical history significant for type 2 diabetes mellitus, treated with Metformin, and small cell carcinoma of the bladder, treated with radical cystectomy and orthotopic ileal neobladder, radiotherapy was not performed. In 2003 he was diagnosed an incisional hernia and underwent open prosthetic repair with a composite mesh (Composix™ E/X Mesh, Bard) implanted in intraperitoneal position.

**Fig. 1:**
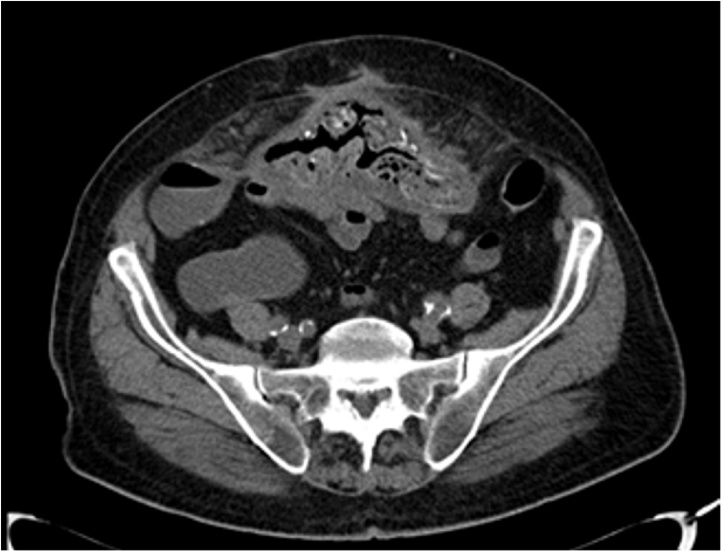
Preoperative CT scan demonstrating small bowel loops adherent to the mesh.

**Fig. 2:**
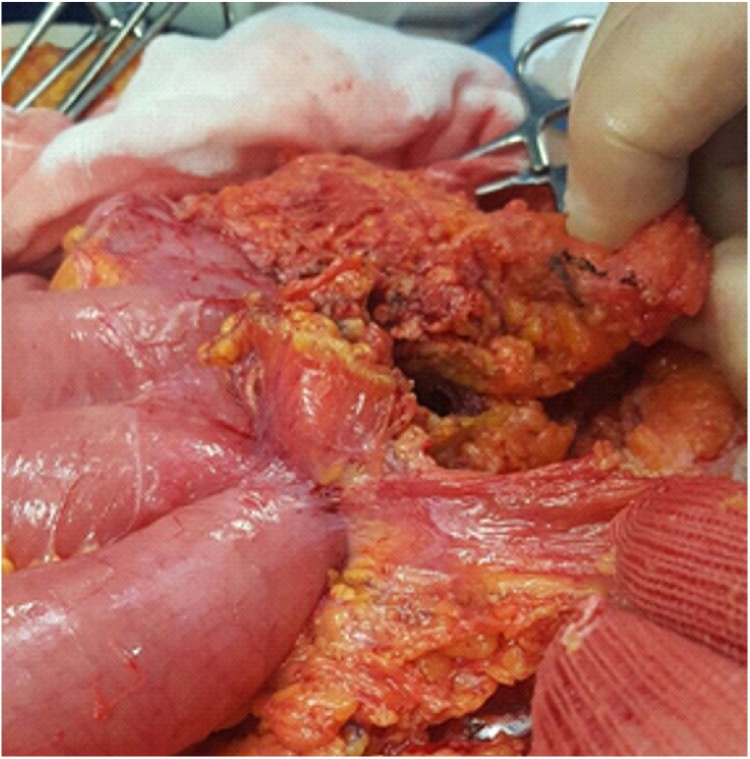
At laparotomy, evidence of adherent ileal loops forming a large mass.

**Fig. 3:**
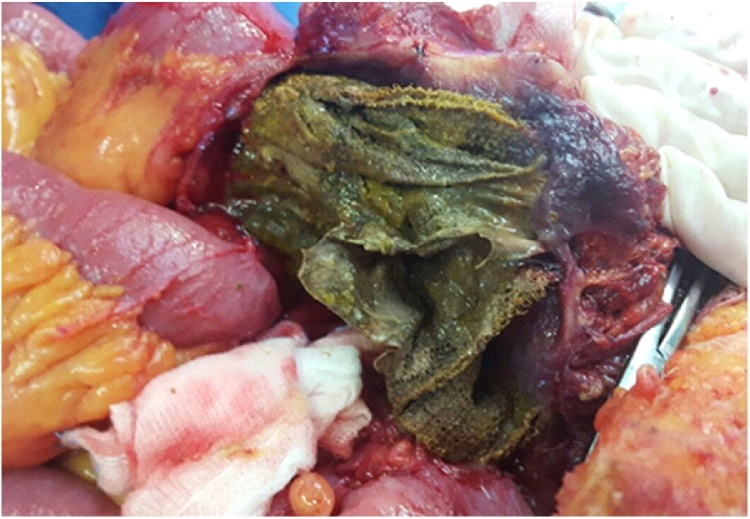
Evidence of the mesh completely intraluminal.

At admission to our Unit, a physical exam showed a well-healed midline laparotomy incision with no evidence of hernia. As completion to previous endoscopic procedures, a CT scan was performed showing entero-enteric fistulae and migration of prosthesis into adherent intestinal loops (Fig. 1, Fig. 2). Informed consent had been previously given by the patient who was treated by a high volume experienced surgeon. The patients underwent laparotomy and a large mass of about 25 cm of diameter, consisting of adherent ileal loops, was found (Fig. 3). Prosthetic material penetrating the bowel was detected, resulting in a natural by-pass between the intestinal loops, which explained the absence of canalization-related symptoms. A dual intestinal resection was performed. An inflammatory process involving the rectus muscles made the abdominal wall repair very challenging. A 30 × 30 cm absorbable mesh (Vycril^®^-Ethicon) was used to reconstruct the posterior fascia of the rectus muscles. A transversus abdominis release (TAR) could not be used due to the critical conditions of the posterior components of the abdominal wall.

According to VHWG grade III, an appropriately shaped, not cross-linked, 20 × 30 cm biological implant (SurgiMend^®^, Integra LifeScience) was positioned in the retromuscular site. A Prevena™ Incisional System (KCI) was used to protect the skin and removed after 6 days. A small dehiscence of the lower third of the surgical wound was found and treated with V.A.C.^®^ Therapy (KCI) for one week, and then with advanced wound care. The patient was discharged on the 23rd postoperative day. The 3-month clinical examination showed the surgical wound well healed. After 9 months the patient is still in good health, with complete resolution of previous anemia.

## 3 Discussion

Prosthetic surgery is actually the gold standard in ventral hernia repair. The use of meshes, especially if placed in direct contact with the viscera, can cause significant morbidity. Mesh-related visceral complications (MRVC), can appear over a period of several years, making the diagnosis very challenging. Recently, an increasing number of cases of mesh adhesion, erosion and migration into hollow organs has been reported in the literature, suggesting the appearance of a new pathology, though the complete intraluminal migration remains a very uncommon event.

Causes of mesh migration are still not clear. Two different mechanisms have been hypothesized, a primary mechanical migration and a secondary migration [3,[5], [6], [7]]. The first one, depending either on inadequate fixation to the fascia or adequate fixation complicated by sliding via external forces, would result in the displacement of the mesh into contiguous anatomical spaces. Later, the mesh could erode into adjacent tissue. Secondary migration might instead occur through trans-anatomical planes, as the result of foreign body reaction. This mechanism is supported by the presence of inflammatory granulation tissue at the site of migration and it is dependent to the extent and nature of the prosthetic material. This process is slow and may require several years. Some authors have assumed that it might develop when a part of the bowel is accidentally included in the fixation of the mesh [8]. Another theory advocates that the presence of adhesions, due to previous hernia repair, might predispose the patient to further adhesion-formation and subsequently lead to migration [9]. The cut edges of the mesh could damage the surface of surrounding organs and provoke an inflammatory response leading to weakness and erosion [10].

None of these theories would fully explain the outcome of the reported patient, having had a peculiar evolution as it presented as microcytic chronic anemia of unknown origin 14 years after open prosthetic incisional hernia repair, without evidence of chronic infection (thrombocytosis, elevated CRP), blind loop with breath testing or short gut with other markers of malnutrition. A case of transmural mesh migration after laparoscopic abdominal wall repair, showing a similar unusual presentation was recently published [11]. Both these reports highlight that anemia was likely due to chronic blood loss from the ulcerated bowel where the mesh eroded through the wall. Thus migration should be considered as a possible etiology of anemia in patients after mesh hernia repair especially if the mesh had been placed underlay.

The earliest report of migration was published by Herrera [12] in 1976, followed by another case, the migration of a wire from a stainless steel wire mesh leading to intestinal obstruction and chronic anemia after 30 years from the repair of a ventral hernia [13]. Six more cases of intraluminal mesh were reported, causing either fistulas [14,15], abdominal pain [5,16,17] or obstruction [11,18]. One case associated with chronic anemia [11] has been published. Picchio et al. recently conducted a MEDLINE search, founding 11 significant cases and reporting a personal case of transmural mesh migration from the abdominal wall into the small bowel, that caused recurrent small bowel obstruction, 18 years after multiple previous attempts of ventral hernia repair [19].

The growing number of reports of MRVC has elicited greater concern in recent years about the placement of prosthetic material in direct contact with the viscera, due to the increase of the risk of adhesions, erosions, and fistula formation. The choice of the proper prosthetic material, suitable to minimize adhesion-formation would be elemental. Manufacturers continuously develop new meshes recommended for intraperitoneal use but their effectiveness is unproven at the moment and it’s still far from the ideal prosthesis [20].

Composite meshes are particularly designed for intraperitoneal placement, with an absorbable or permanent barrier on the internal side to reduce adhesion formation. In our patient, a Composix™ E/X Mesh (Bard) was implanted as reinforcement in the previous abdominal wall repair. In this prosthesis, the inner e-PTFE barrier should decrease the risk of adhesions. Adhesions still developed, leading to erosion, followed by mesh migration. In our experimental study, we demonstrated that prostheses manufactured for intraperitoneal use decrease, but do not eliminate, adhesion formation and consequent complications [20]. For this reason, either a natural barrier, as omentum, peritoneum or the posterior rectus sheath, or an absorbable material, should be interposed between the mesh and the underlying viscera. In our case, an absorbable Vycril^®^ layer was placed as temporary barrier between the biologic implant (SurgiMend^®^, Integra LifeScience) and viscera below, to reconstruct the posterior rectus sheath and minimise adhesion formation.

Our patient had an unusual presentation as he complained non-specific persistent pain and indefinite gastro-intestinal symptoms with chronic microcytic anemia, similar to the case described by Voisard and Feldman [11].

## 4 Conclusion

Prosthetic abdominal wall repair may result in significant mesh related specific complications.

Mesh migration and erosion, are uncommon complications of prosthetic incisional hernia repair. They may occur also several years after abdominal wall repair and should be considered in atypical patient presentations. We here report a case of migration of a composite mesh, 14 years after a prosthetic incisional hernia repair. The peculiarity of this case lies in the unusual presentation with persistent abdominal pain and microcytic anemia. This is a reminder that in patients complaining abdominal pain and chronic microcytic anemia after previous prosthetic abdominal wall repair, mesh-related complications, as mesh erosion and possible migration, should be considered as possible etiology, when all other causes of GI symptoms have been ruled out.

## Conflicts of interest

All authors declare that there are no financial and personal relationships with other people or organisations that could inappropriately influence their work.

## Sources of funding

All authors declare that any funding from any source has been received to perform the study.

## Ethical approval

No ethical approval is requested because we are submitting a Case Report and not a research study.

Consequently, no ethical approval from any committee has been submitted.

## Consent

Written informed consent was obtained from the patient for publication of this case report and accompanying images. A copy of the written consent is available for review by the Editor-in-Chief of this journal on request.

## Author contributions

Ceci Francesca M.D.: Conception and design of the work, data analysis and interpretation, drafting the paper.

D’Amore Linda M.D.: Drafting the paper, data interpretation Annesi Elena M.D.: data collection.

Bambi Lucia M.D.: Data collection.

Grimaldi Maria Romana M.D.: Data collection Gossetti Francesco M.D.: data interpretation.

Negro Paolo M.D.: Data interpretation, critical revision of the paper, final approval of the version to be published.

## Registration of research studies

No registration is requested.

## Guarantor

Francesca Ceci, MD, PhD.

## Provenance and peer review

Commissioned, externally peer-reviewed.

## References

[1] Agha R.A., Fowler A.J., Saetta A., Barai I., Rajmohan S., Orgill D.P., for the SCARE Group (2016). The SCARE statement: consensus-based surgical case report guidelines. Int. J. Surg..

[2] Mudge M., Hughes L.E. (1985). Incisional hernia: a 10-year prospective study of incidence and attitudes. Br. J. Surg..

[3] Kingsnorth A., LeBlanc K. (2003). Hernias: inguinal and incisional. Lancet.

[4] Conze J., Binnebosel M., Junge K., Schumpelick V. (2010). Incisional hernia. How do I do it? Standard surgical approach. Chirurg.

[5] Gandhi D., Marcin S., Xin Z., Asha B., Kaswala D., Zamir B. (2011). Chronic abdominal pain secondary to mesh erosion into cecum following incisional hernia repair: a case report and literature review. Ann. Gastroenterol..

[6] Agrawal A., Avill R. (2006). Mesh migration following repair of inguinal hernia: a case report and review of literature. Hernia.

[7] Jha A.K., Nijhawan S., Pokharna R., Nepalia S., Suchismita A. (2012). Colo-cutaneous fistula formation due to delayed mesh migration following lumbar hernia repair: colonoscopic diagnosis. Trop. Gastroenterol..

[8] Celik A., Kutun S., Kockar C., Mengi N., Ulucanlar H., Cetin A. (2005). Colonoscopic removal of inguinal hernia mesh: report of a case and literature review. J. Laparoendosc. Adv. Surg. Tech. A.

[9] Goswami R., Babor M., Ojo A. (2007). Mesh erosion into caecum following laparoscopic repair of inguinal hernia (TAPP): a case report and literature review. J. Laparoendosc. Adv. Surg. Tech. A.

[10] Riaz A.A., Ismail M., Barsam A., Bunce C.J. (2004). Mesh erosion into the bladder: a late complication of incisional hernia repair. A case report and review of the literature. Hernia.

[11] Voisard G., Feldman L.S. (2013). An unusual cause of chronic anemia and abdominal pain caused by transmural mesh migration in the small bowel after laparoscopic incisional hernia repair. Hernia.

[12] Herrera M.A., Hsia T.W., Becker D.R. (1976). Migration of teflon mesh from abdominal wall into large bowel. N. Y. State J. Med..

[13] Majeski J. (1998). Migration of wire mesh into the intestinal lumen causing an intestinal obstruction 30 years after repair of a ventral hernia. South. Med. J..

[14] Horzic M., Vergles D., Cupurdija K., Kopljar M., Zidak M., Lackovic Z. (2011). Spontaneous mesh evacuation per rectum after incisional hernia repair. Hernia.

[15] Nelson E.C., Vidovszky T.J. (2011). Composite mesh migration into the sigmoid colon following ventral hernia repair. Hernia.

[16] Yilmaz I., Karakaş D.O., Sucullu I., Ozdemir Y., Yucel E. (2013). A rare cause of mechanical bowel obstruction: mesh migration. Hernia.

[17] Millas S.G., Mesar T., Patel R.J. (2015). Chronic abdominal pain after ventral hernia due to mesh migration and erosion into the sigmoid colon from a distant site: a case report and review of literature. Hernia.

[18] Steinhagen E., Khaitov S., Steinhagen R.M. (2010). Intraluminal migration of mesh following incisional hernia repair. Hernia.

[19] Picchio M., Muggianu A., Mancini F., Tintisona O., Spaziani E. (2017). Complete mesh migration into the small bowel after incisional hernia repair: a case report and literature review. Acta Chir. Belg..

[20] D’Amore L., Ceci F., Mattia S., Fabbi M., Negro P., Gossetti F. (2017). Adhesion prevention in ventral hernia repair: an experimental study comparing three lightweight porous meshes recommended for intraperitoneal use. Hernia.

